# Ear Structures of the Naked Mole-Rat, *Heterocephalus glaber*, and Its Relatives (Rodentia: Bathyergidae)

**DOI:** 10.1371/journal.pone.0167079

**Published:** 2016-12-07

**Authors:** Matthew J. Mason, Hannah L. Cornwall, Ewan St. J. Smith

**Affiliations:** 1 University of Cambridge, Department of Physiology, Development & Neuroscience, Cambridge, United Kingdom; 2 University of Cambridge, Department of Pharmacology, Cambridge, United Kingdom; University of Pretoria, SOUTH AFRICA

## Abstract

Although increasingly popular as a laboratory species, very little is known about the peripheral auditory system of the naked mole-rat, *Heterocephalus glaber*. In this study, middle and inner ears of naked mole-rats of a range of ages were examined using micro-computed tomography and dissection. The ears of five other bathyergid species (*Bathyergus suillus*, *Cryptomys hottentotus*, *Fukomys micklemi*, *Georychus capensis* and *Heliophobius argenteocinereus*) were examined for comparative purposes. The middle ears of bathyergids show features commonly found in other members of the Ctenohystrica rodent clade, including a fused malleus and incus, a synovial stapedio-vestibular articulation and the loss of the stapedius muscle. *Heterocephalus* deviates morphologically from the other bathyergids examined in that it has a more complex mastoid cavity structure, poorly-ossified processes of the malleus and incus, a ‘columelliform’ stapes and fewer cochlear turns. Bathyergids have semicircular canals with unusually wide diameters relative to their radii of curvature. How the lateral semicircular canal reaches the vestibule differs between species. *Heterocephalus* has much more limited high-frequency hearing than would be predicted from its small ear structures. The spongy bone forming its ossicular processes, the weak incudo-stapedial articulation, the columelliform stapes and (compared to other bathyergids) reduced cochlear coiling are all potentially degenerate features which might reflect a lack of selective pressure on its peripheral auditory system. Substantial intraspecific differences were found in certain middle and inner ear structures, which might also result from relaxed selective pressures. However, such interpretations must be treated with caution in the absence of experimental evidence.

## Introduction

Bathyergid mole-rats, endemic to Africa, are members of the Ctenohystrica clade of rodents, a group which also contains the caviomorphs of South America [1]. The most famous bathyergid must be the eusocial naked mole-rat, *Heterocephalus glaber*. *Heterocephalus* lives in underground colonies of, on average, 75–80 individuals, among which reproduction is usually restricted to just one female and one to two males (see [2] for a review). Naked mole-rats are weaned at around 3–6 weeks of age, can become reproductively active at 7–12 months, reach full adult size at about 2 years and can live for more than 28 years (reviewed by [3]).

Visual cues are very limited in mole-rat burrows. The small eyes of naked mole-rats can apparently detect the difference between light and dark [4], as can the eyes of other bathyergids [5], but they are ill-equipped for image formation. There are challenges to audition underground too: airborne sound, particularly at high frequencies, has been shown to be rapidly attenuated with distance within the burrow systems of another mole-rat [6]. Low frequencies of a few hundred Hertz propagate most effectively. It has been suggested that certain frequencies may be selectively amplified in a “stethoscope effect” [7].

Despite the limits to sound propagation underground, vocalisations appear to be important in the social communication of *Heterocephalus*. The naked mole-rat has an extensive repertoire of at least 17 distinct sounds, including five juvenile-only vocalisations. Many are shorter than 200 ms duration but most are repeated. Atonal sounds contain frequencies from 0.2 to over 40 kHz, while tonal sounds fall between 1 and 9 kHz. Naked mole-rats can also scrape their upper and lower incisors together to generate click-like sounds with most energy between 1 and 20 kHz, but it is not known if these sounds are used to communicate [8].

The hearing of *Heterocephalus* has been examined behaviourally, using a conditioned avoidance method [9]. For airborne sound presented at 60 dB SPL, the hearing range of this animal extends between 65 Hz and 12.8 kHz, which is an extremely restricted, low-frequency range for a small mammal. Thresholds between 125 Hz and 8 kHz are rather flat and unusually high, the lowest being 35 dB SPL. Together with its very poor sound localization ability, these characteristics led Heffner & Heffner [9] to dub the hearing of the naked mole-rat “degenerate”. Heffner & Heffner suggested that the limitations on hearing in this animal are central rather than peripheral, based on the fact that auditory thresholds are reduced with increased sound duration. A recent examination of central auditory structures in the naked mole-rat has shown that the binaural auditory brainstem nuclei lack the ion channel HCN1 [10]. This could result in slowed integration of inputs in binaural auditory brainstem neurons, which could impede the analysis of interaural time differences, potentially contributing to poor localization abilities [10]. Insensitive hearing restricted to low frequencies is also a feature of a Zambian bathyergid originally referred to as *Cryptomys* sp. but subsequently reidentified as *Fukomys anselli* [11, 12], and has also been found in the more distantly-related spalacid mole-rat *Spalax* [13, 14] and the pocket gopher *Geomys* [15]. It appears to be a general feature of subterranean rodents. The somewhat wider hearing range of the coruro *Spalacopus*, a fossorial caviomorph, is thought to relate to an increased amount of above-ground behaviour [16].

The fact that underground tunnels represent a ‘low-frequency’ environment has been used to explain why vocalizations of subterranean rodents tend to emphasize such frequencies [6, 16–19]. This suggests that the peripheral auditory systems of these animals might show convergently-derived, low-frequency adaptations, and many similarities have indeed been found between the ear structures of distantly-related groups. Most subterranean mammals have ‘freely mobile’ ossicles which are relatively loosely articulated with the skull, reduced or missing middle ear muscles, relatively large stapes footplates and tympanic membranes lacking a pars flaccida [20–24]. Freely-mobile ossicles may improve low-frequency sound transmission through their reduced stiffness, but the physiological significance of the other features remains unclear. The anatomical characteristic most closely linked to improved low-frequency hearing in small mammals seems to be an enlarged middle ear cavity volume [25], but subterranean species do not, in general, have markedly expanded middle ear cavities compared to terrestrial species [21]. It has been argued that, despite the selective pressure towards low-frequency hearing, hearing sensitivity need not be enhanced in these animals because of the so-called “stethoscope effect” operating in their tunnels; the notion that the hearing of these animals is degenerate has been strongly rejected [7, 20].

Among bathyergids, the middle and inner ears of species in the *Cryptomys*/*Fukomys* clade have been examined in detail [26–35]. The ears of other bathyergids have received much less attention, although brief descriptions exist of the middle ear apparatus in *Bathyergus*, *Georychus* [20] and *Heliophobius* [36, 37]. The lack of information on *Heterocephalus* is surprising: the only description of the middle ear of the naked mole-rat which we are aware of comes from the unpublished PhD thesis of one of the present authors [38], and we have found no information in the literature relating to its inner ear.

The purpose of the current paper is to produce the first detailed anatomical description of the peripheral auditory system of *Heterocephalus*, considering not just adult morphology but also postnatal ontogeny. *Heterocephalus* is increasingly used as a laboratory model species in research into cancer, ageing and pain [39]. This description of its auditory apparatus complements the increasing body of knowledge being gathered about this strange and enigmatic mammal, and allows us to investigate the extent to which its peripheral auditory system contributes towards its restricted hearing. Other bathyergid species were examined for comparative purposes: this is the first ear study to include representatives of all six currently-recognised bathyergid genera.

## Materials and Methods

15 corpses of *Heterocephalus glaber*, all non-breeders, were obtained from a laboratory colony housed in the University of Cambridge (Table 1); most were preserved frozen until use. As detailed in Table 1, these animals were either members of the original Cambridge colony or offspring of these original members. The first Cambridge colony was formed of animals taken from another captive colony, but data on the number of generations bred there were unfortunately unavailable. The four youngest mole-rats used in this study had died naturally, perhaps as a result of maternal neglect. The older animals had been sacrificed and in most cases their brains removed as part of another research project, which sometimes resulted in damage to the ear regions. Although most specimens were obtained unfixed, two of the adult *Heterocephalus* had been anaesthetised with pentobarbitone and transcardially perfused with phosphate-buffered saline (pH 7.4), followed by 4% paraformaldehyde (at around pH 7.4). All experiments on *Heterocephalus* had been conducted in accordance with the United Kingdom Animal (Scientific Procedures) Act 1986 under a Project License (70/7705) granted to E.S.J.S. by the Home Office; the University of Cambridge Animal Welfare Ethical Review Body also approved procedures. A further three newborn *Heterocephalus* specimens, all from the same litter, had been obtained in 1997 as perinatal casualties from a colony in Bristol Zoo and stored frozen. One of these had been histologically sectioned. Having been left in Shandon Formal-Fixx fixative (10% neutral buffered formalin) for six days, it was put into Shandon standard decalifier for three days. Following dehydration and embedding in paraffin wax, sections were taken at 10 μm thickness and stained with haematoxylin and eosin.

**Table 1:** Details of specimens examined in this project, including CT scan information.

Species	Spec. No.	Age, days	Litter	Body mass, g	Sex	Scan made	Scanned	Voxel side length, μm
*Heterocephalus glaber*	CU1	1166	B1	44.5	M	Cambridge	Bulla	5.4
	CU2	1316	A1	54.5	M	-	-	-
	CU3	1358	A1	63.5	M	Cambridge	Head	17.8
	CU4	1	C3	1.8	F	Cambridge	Head	7.5
	CU5	1656	A1	43.5	M	Cambridge	Head	15.7
							Bulla	6.9
	CU7	1753	A1	58.0	M	-	-	-
	CU8	37	D3	2.3	M	Cambridge	Head	7.8
	CU9	37	D3	1.1	M	-	-	-
	CU10	2	E2	1.0	?	-	-	-
	CU15	204	D3	44.5	F	-	-	-
	CU16	212	D3	38.5	M	-	-	-
	CU17	194	D3	38.0	M	Cambridge	Bullae	6.0
	CU18	176	D3	36.0	F	Cambridge	Bulla	4.8
	CU19	1997	A1	81.5	M	Cambridge	Head	16.8
							Bulla	6.1
	CU20	118	F3	23.0	M	Cambridge	Head	13.6
							Bulla	4.9
	BZ3	?1	G	1.4	?	-	-	-
	BZ4	?1	G	1.1	M	-	-	-
	BZ5	?1	G	1.5	M	Cambridge	Head	7.6
*Bathyergus suillus*	178	?	N/A	?	?	Hull	Skull	48.4
	618	?	N/A	650	M	Hull	Skull	45.1
*Cryptomys hottentotus*	E3116	?	N/A	?	?	Cambridge	Skull	12.6
	E3118	?	N/A	?	?	Cambridge	Skull	13.3
	E3119	?	N/A	?	?	Cambridge	Skull	15.3
*Fukomys micklemi*	F1	?	N/A	?	F	Cambridge	Head	21.5
							Bulla	6.8
	F2	?	N/A	?	M	Cambridge	Head	22.5
*Georychus capensis*	E3110	?	N/A	?	?	Cambridge	Skull	21.2
*Heliophobius argenteocinereus*	Ha25	?	N/A	198	F	Hull	Skull	30.9
	Ha38	?	N/A	148	F	Hull	Skull	33.2

*Heterocephalus* ‘CU’ specimens were from the colony in the University of Cambridge while ‘BZ’ specimens came originally from Bristol Zoo. The litter lettering indicates which naked mole-rats were siblings. The associated number 1 refers to a member of the original Cambridge colony, 2 to an animal bred from the colony queen, and 3 to animals bred from a male and female which had been separated from the original colony. CT scans from the University of Hull were made by Dr. Phil Cox as part of a separate research project.

Dissections of the *Heterocephalus* specimens were performed using a Motic binocular light microscope fitted with a GX-CAM 5 digital camera. GXCapture 8.0 software (GT Vision) was used to take images and make scaled measurements from them. Light microscope images were merged using Helicon Focus 4.10 (Helicon Soft Ltd., 2007), to increase apparent depth of focus. Dissection and CT images originally of right ear structures were laterally inverted, to facilitate comparison.

Three skulls of *Cryptomys hottentotus* (UMZ E3116, E3118 and E3119, originally collected in Pietermaritzburg, South Africa) and one skull of *Georychus capensis* (UMZ E3110, originally collected at the Cape of Good Hope, South Africa), from the collections of the University Museum of Zoology, Cambridge, were CT-scanned.

Two heads of *Fukomys micklemi*, formalin-preserved and stained with iodine for use in another project, were donated by Dr. Phil Cox. The specimens had originally been obtained from a colony at the University of Ghent, belonging to Prof. Dominique Adriaens. Dr. Cox also kindly provided us with computed tomograms of prepared skulls of *Bathyergus suillus* and *Heliophobius argenteocinereus*, which had been made at the University of Hull. The two *Bathyergus* specimens had been collected in Cape Town, South Africa, by Prof. Nigel Bennett, while the two *Heliophobius* specimens had been collected in Blantyre, Malawi, by Prof. Radim Šumbera.

### Micro-computed tomography and reconstruction

CT scans were made of intact heads and dissected-out auditory regions of *Heterocephalus* and *Fukomys* specimens, and of the intact, dried skulls of *Cryptomys* and *Georychus* specimens (Table 1). Wet specimens were wrapped in cellophane to minimise drying during the scans, which were all made using a Nikon XT H 225 scanner. The settings used were 125–130 kV and 110–130 μA. Images were constructed from 1080 projections, each with 1000 ms exposure and two frames averaged per projection. The scan data were processed with CT AGENT XT. 3.1.9 and CT PRO 3D XT 3.1.9 (Nikon Metrology, 2004–2013). Cubic voxel side-lengths are given in Table 1.

Tiff stacks from the micro-CT scans were converted to jpegs in Adobe Photoshop CS 8.0 (Adobe Systems Inc., 2003). MicroView 2.5.0 (Parallax Innovations Inc., 2016) was used to produce 3D surface reconstructions and to reorient tomograms. Other reconstructions were made using WinSurf 4.0 (Eric Neufeld, 2001), in a process requiring manual identification of boundaries. The middle ear cavity reconstructions were based on its internal boundaries, while inner ear reconstructions followed the inner walls of the bony labyrinth. Cartilage, soft tissue and fluid were impossible to distinguish from each other in the CT scans, which made it difficult to identify some of the structural boundaries in the neonatal *Heterocephalus* specimens, prior to ossification. The formalin-preserved *Fukomys* specimens proved to have undergone some demineralisation and crystallisation within the auditory bullae, but it was possible to distinguish and model the ear structures. Each recorded measurement was obtained from one ear only per animal.

In order to make cochlear measurements, tomograms were reoriented in MicroView such that the *z*-axis ran through the centre of the modiolus. The 3D coordinates of multiple points along the centre of the cochlear canal, starting from the beginning of the primary spiral lamina near the round window and extending to the apex, were then recorded. At least one point was recorded every 10° along the spiral. The total length of the cochlear canal was estimated as the sum of the linear distances between consecutive points. The number of spiral turns and the radii of curvature at base (*R*_base_) and apex (*R*_apex_) were established from the *x-y* coordinates of these points. The radii of curvature were calculated following the method of Manoussaki et al. [40], except that, for simplicity, the five points used were separated by 22.5 degree steps from the estimated centre of the cochlear spiral, rather than being equally spaced. Measurements of semicircular canal radii of curvature were derived from measurements of the ‘height’ and ‘width’ of the arcs, following Ekdale [41]. The internal diameter of each bony semicircular canal was calculated as the mean of two orthogonal measurements, made at the point on the canal arc where the ‘height’ measurement was taken.

Because they were originally made for the purposes of another project, the scans of the *Bathyergus* and *Heliophobius* specimens were at a lower resolution than those of the other species. Semicircular canal measurements were not made because the resolution was insufficient. The descriptions of the inner ear and ossicles in these animals should be considered less reliable than those of the other species.

## Results

The anatomical description in the first two sub-sections below is restricted to older *Heterocephalus* specimens (>100 days) unless otherwise noted. The ears of the younger specimens are described in the third sub-section, and the ears of other bathyergids in the fourth. Measurement data are presented in the tables below; additional data are presented as supporting information (see S1 Table).

### The external and middle ears of *Heterocephalus*

**Fig 1:**
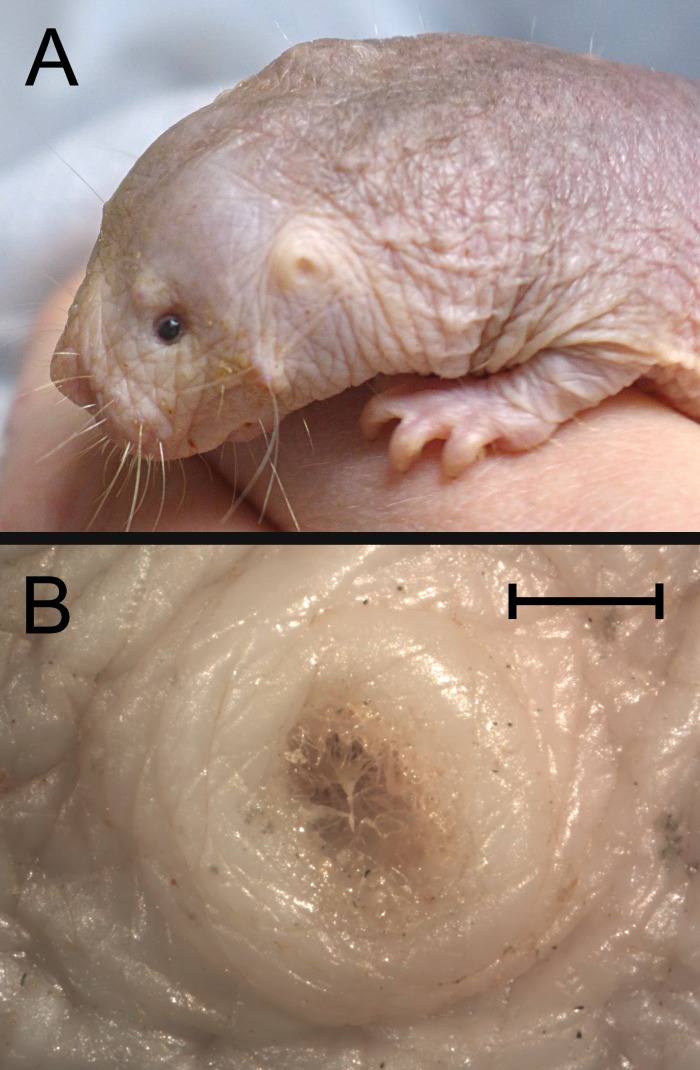
The external ear of *Heterocephalus*. A: Lateral view of a live, adult animal. The position of the external ear, which lacks a pinna, is indicated by an arrow. B: Photomicrographic close-up of the left external ear opening of a different adult specimen, post-mortem. Note that the lumen is semi-occluded with hairs and cerumen. Scale bar for B is 1 mm.

The naked mole-rat has no pinnae; the only sign of the external ear on the surface of the head is a raised annulus (Fig 1). The entrance to the meatus in the centre of this annulus was typically semi-occluded with fine hairs and a small amount of cerumen. The meatus projects ventromedially into the head to reach the auditory bulla. It is around 6 mm long with a very narrow but patent lumen, diameter around 0.6 mm. Away from the surface of the head, the meatus is largely free of hairs and cerumen.

**Fig 2:**
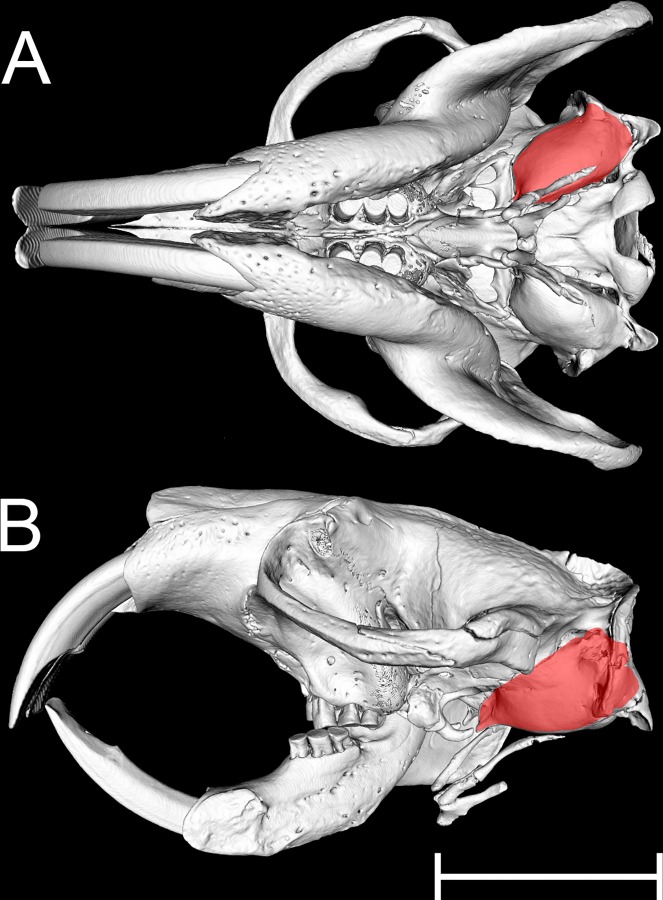
MicroView reconstruction of the skull of an adult *Heterocephalus* (65 months). A: Ventral view of skull. B: Lateral view, the left mandible having been digitally removed. The approximate extent of the left middle ear cavity is indicated in both cases by red shading. Scale bar 10 mm.

The auditory bulla of *Heterocephalus* is relatively small (Fig 2). The walls of the middle ear cavity were well-ossified in the adult specimens, in which tympanic and petrosal components were indistinguishably fused.

**Fig 3:**
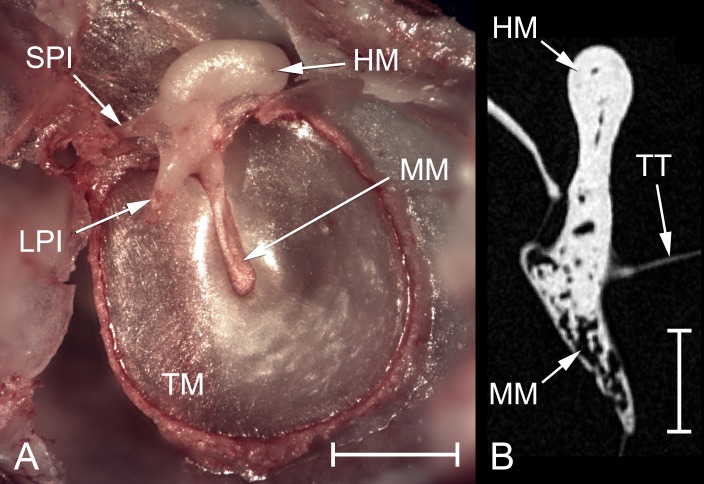
Middle ear structures of *Heterocephalus*. A: Photomicrograph of a dissected left ear of an adult *Heterocephalus* (54 months), medial view. The malleus, incus and tympanic membrane are visible; the stapes and tensor tympani muscle have been removed. Scale bar 1 mm. B: Tomogram section through the left malleus of an adult mole-rat (65 months), posterior view. Note the difference between the compact bone which forms the head and the spongy bone which forms the manubrium. The tensor tympani tendon is also visible. Scale bar 0.5 mm. APSC = ampulla for posterior semicircular canal; ASC = anterior semicircular canal; C = cochlea; DMC = dorsal mastoid cavity; ED = bony tube for endolymphatic duct; ER = epitympanic recess; FP = footplate of stapes; HM = head of malleus; LA = lenticular apophysis of incus; LPI = long process of incus; LSC = lateral semicircular canal; MI = malleoincus; MM = manubrium of malleus; OW = oval window; PC = posterior crus of stapes; PD = bony tube for perilymphatic duct (canaliculus cochleae); PMC = posteromedial mastoid cavity; PSC = posterior semicircular canal; RW = round window; SPI = short process of incus; TC = tympanic cavity; TM = tympanic membrane; TT = tensor tympani tendon; V = vestibule of inner ear; VMC = ventral mastoid cavity.

**Fig 4:**
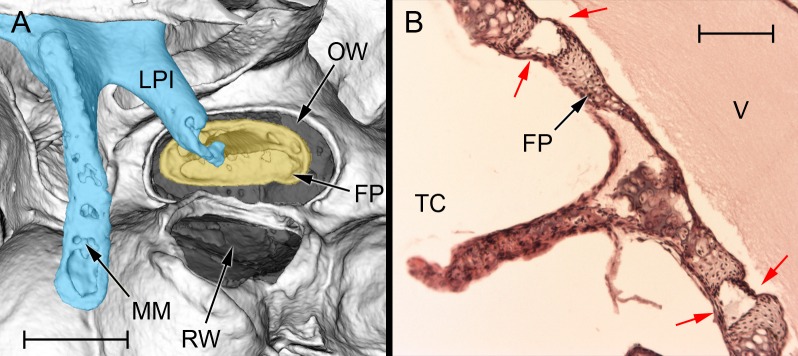
The stapes of *Heterocephalus*, in situ. A: MicroView reconstruction of the left stapes region in an adult mole-rat (65 months), ventrolateral view. The malleoincudal complex has been coloured blue, the stapes yellow. Note the very wide gap between stapes footplate and the rim of the oval window. This gap and the opening of the round window (both shaded grey) are covered over by membranes, but these do not show up on the CT reconstruction. Scale bar 0.5 mm. B: Histological section of the stapes of a neonatal *Heterocephalus*, showing the synovial structure of the stapedio-vestibular articulation. The red arrows indicate the synovial joint capsule, which lies between the stapes footplate and the rim of the oval window. Scale bar 0.1 mm. APSC = ampulla for posterior semicircular canal; ASC = anterior semicircular canal; C = cochlea; DMC = dorsal mastoid cavity; ED = bony tube for endolymphatic duct; ER = epitympanic recess; FP = footplate of stapes; HM = head of malleus; LA = lenticular apophysis of incus; LPI = long process of incus; LSC = lateral semicircular canal; MI = malleoincus; MM = manubrium of malleus; OW = oval window; PC = posterior crus of stapes; PD = bony tube for perilymphatic duct (canaliculus cochleae); PMC = posteromedial mastoid cavity; PSC = posterior semicircular canal; RW = round window; SPI = short process of incus; TC = tympanic cavity; TM = tympanic membrane; TT = tensor tympani tendon; V = vestibule of inner ear; VMC = ventral mastoid cavity.

The middle ear cavity can be divided into tympanic, epitympanic and mastoid components. The tympanic cavity is by far the largest of these (Table 2). A small diverticulum extends laterally from just caudal to the tympanic membrane but otherwise the tympanic cavity is roughly ovoid. The tympanic membrane (Fig 3) forms part of its lateral wall: this averages 4.55 mm^2^ in area (n = 4) and lacks any obvious *pars flaccida*. Within the tympanic cavity, the cochlear promontory forms a rounded prominence medially. There are usually two, very small, partial septa ventral to this. A thin lamina of bone extends from the promontory towards the rostromedial tip of the bulla, just above which is a channel which becomes the Eustachian tube. Dorsal to the promontory, this channel becomes covered over with a thin layer of bone, behind which is located the tensor tympani muscle belly. The tensor tendon emerges posteriorly and inserts on the manubrium of the malleus (Fig 3B). The oval window is just caudal to the muscle belly. There is a strikingly wide gap between the stapes footplate and the oval window perimeter (Fig 4A). The fibrous annular ligament would normally occupy this space, but in the naked mole-rat the stapedio-vestibular articulation was found to be synovial (see later). Immediately below the oval window is the round window, within a shallow recess. No stapedius muscle was found in any specimen of any age.

**Table 2:** Measurements of middle ear subcavity and bony labyrinth volumes.

Species	Spec. No.	Age, days	ER volume, mm^3^	TC volume, mm^3^	VMC volume, mm^3^	DMC volume, mm^3^	PMC volume, mm^3^	Bony labyrinth volume, mm^3^
*Heterocephalus glaber*	CU4	1	0.51	5.42	0	0	0	3.91
	BZ5	?1	0.65	7.20	0	0	0	4.48
	CU8	37	0.60	5.79	0	0	0	4.02
	CU20	118	1.47	16.76	3.08	4.14	0.35	4.22
	CU17	194	1.74	(damaged)	6.23	6.93	0.83	(damaged)
	CU1	1166	1.57	18.33	3.59	1.81	0.14	3.56
	CU5	1656	1.99	21.06	4.88	(damaged)	(damaged)	(damaged)
	CU19	1997	1.70	17.37	5.44	4.66	0.46	3.53
*Bathyergus suillus*	178	?	111.66	318.63		7.41	0	17.79
	618	?	112.16	322.69		4.89	0	18.29
*Cryptomys hottentotus*	E3116	?	9.92	48.74		2.19	0	5.01
	E3118	?	14.39	48.27		2.85	0	6.87
	E3119	?	12.84	43.32		1.97	0	6.88
*Fukomys micklemi*	F1	?	19.28	74.16		0.83	0	7.60
	F2	?	19.50	75.31		1.27	0	N/A
*Georychus capensis*	E3110	?	25.37	101.53		1.77	0	9.35
*Heliophobius argenteocinereus*	Ha25	?	24.16	79.45		2.30	0	9.20
	Ha38	?	33.62	99.04		4.06	0	8.53

Note that the ventral mastoid cavity (VMC) could be distinguished from the tympanic cavity only in *Heterocephalus*, and only this species has a posteromedial mastoid cavity (PMC). In some *Heterocephalus* specimens, ear structures were damaged and some measurements therefore could not be made. In the very young specimens the outlines of the cavities were sometimes very unclear, so these values should be considered estimates.

Dorsal to the tympanic cavity is the roughly pyramidal epitympanic recess, containing the bodies of the fused malleus and incus. The manubrium of the malleus and the incudal long process extend through the small, triangular opening between the two cavities. The short process of the incus projects into a blind-ending, posterior diverticulum of the epitympanic recess, where it is attached to the skull by means of ligamentous material inserting around its tip. The facial nerve passes just under the tip of the short process, in a bony canal which is open to the middle ear cavity over some of its length.

**Fig 5:**
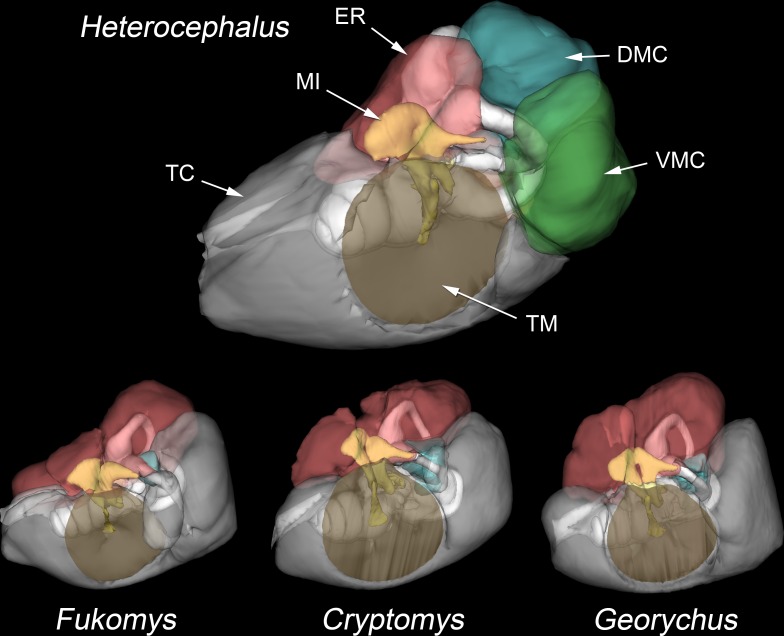
Middle ear cavities of bathyergids. WinSurf reconstructions of the left middle ear cavities and associated structures in four species of bathyergids (*Heterocephalus glaber*, *Fukomys micklemi*, *Cryptomys hottentotus* and *Georychus capensis*), from lateral and slightly anterior views. The walls of the middle ear cavities are shown semitranslucent. Positions of the tympanic membranes are indicated by brown shading. The epitympanic recess is colour-coded red, the dorsal mastoid cavity blue and the ventral mastoid cavity green. The ossicles are yellow. The posteromedial mastoid cavity, present only in *Heterocephalus*, is not visible in this view. The posterior parts of the middle ear cavities of the other species extend into the mastoid region and are equivalent to the ventral mastoid cavity of *Heterocephalus*, but they lack constricted entrances and so have not been separately coloured. Not to scale. APSC = ampulla for posterior semicircular canal; ASC = anterior semicircular canal; C = cochlea; DMC = dorsal mastoid cavity; ED = bony tube for endolymphatic duct; ER = epitympanic recess; FP = footplate of stapes; HM = head of malleus; LA = lenticular apophysis of incus; LPI = long process of incus; LSC = lateral semicircular canal; MI = malleoincus; MM = manubrium of malleus; OW = oval window; PC = posterior crus of stapes; PD = bony tube for perilymphatic duct (canaliculus cochleae); PMC = posteromedial mastoid cavity; PSC = posterior semicircular canal; RW = round window; SPI = short process of incus; TC = tympanic cavity; TM = tympanic membrane; TT = tensor tympani tendon; V = vestibule of inner ear; VMC = ventral mastoid cavity.

**Fig 6:**
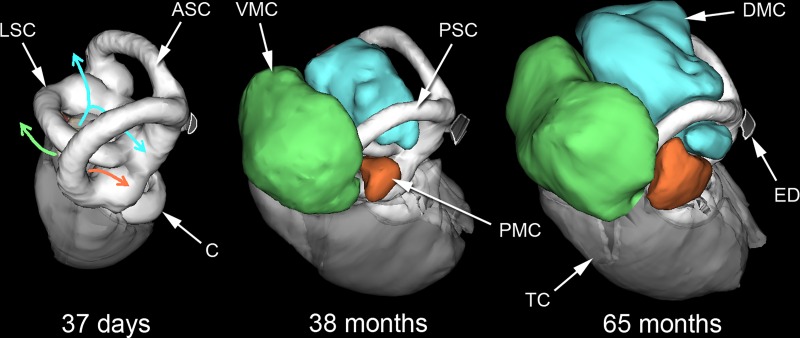
Bony labyrinths and mastoid cavities of *Heterocephalus*. WinSurf reconstructions of the left bony labyrinths and mastoid cavities of three *Heterocephalus* specimens of different ages, seen from approximately posterior views. In the youngest specimen on the left, coloured arrows indicate the future directions of expansion of the middle ear cavity into the mastoid region. Blue = dorsal mastoid cavity; green = ventral mastoid cavity; orange = posteromedial mastoid cavity. The 38-month-old specimen had smaller mastoid cavities than many younger specimens, so this diagram should not be taken to indicate a strict temporal sequence. APSC = ampulla for posterior semicircular canal; ASC = anterior semicircular canal; C = cochlea; DMC = dorsal mastoid cavity; ED = bony tube for endolymphatic duct; ER = epitympanic recess; FP = footplate of stapes; HM = head of malleus; LA = lenticular apophysis of incus; LPI = long process of incus; LSC = lateral semicircular canal; MI = malleoincus; MM = manubrium of malleus; OW = oval window; PC = posterior crus of stapes; PD = bony tube for perilymphatic duct (canaliculus cochleae); PMC = posteromedial mastoid cavity; PSC = posterior semicircular canal; RW = round window; SPI = short process of incus; TC = tympanic cavity; TM = tympanic membrane; TT = tensor tympani tendon; V = vestibule of inner ear; VMC = ventral mastoid cavity.

Three subcavities separately extend from the posterior part of the tympanic cavity into the mastoid region of the skull (Figs 5 and 6). Two of them show a similar morphology to cavities described in the gerbil *Meriones unguiculatus* [42], and so the same nomenclature has been adopted. The dorsal mastoid cavity (DMC) emerges through the window framed by the lateral semicircular canal and occupies most of the space between the three canals. The entrance to the ventral mastoid cavity (VMC) is framed by the lateral and posterior semicircular canals, the canal for the facial nerve and a partial septum dividing it from the tympanic cavity. The VMC was internally divided by a septum in one 54-month animal. The posteromedial mastoid cavity (PMC; not described in *Meriones*) is the smallest of the subcavities. It emerges through a narrow gap left between the lateral and posterior semicircular canals (Fig 6). These three mastoid subcavities appear to ‘jostle for position’ around the semicircular canals and there were individual differences in their relative shapes and sizes. However, there was no clear relationship between age and the volumes of the middle ear subcavities in specimens older than 100 days (Table 2).

**Fig 7:**
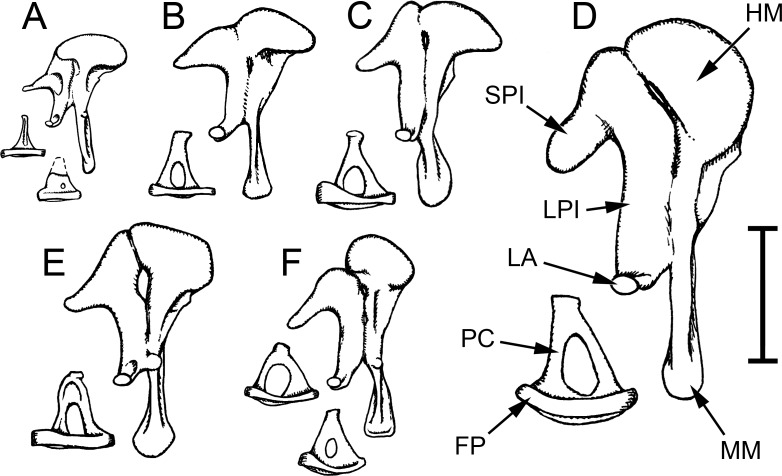
Auditory ossicles of bathyergids. Left auditory ossicles of bathyergid mole-rats, drawn to scale. The mallei and incudes, which are fused in these animals, are shown from an internal view in each case, the stapedes from a dorsal view. A: *Heterocephalus glaber*; B: *Fukomys micklemi*; C: *Heliophobius argenteocinereus*; D: *Bathyergus suillus*; E: *Georychus capensis*; F: *Cryptomys hottentotus*. Two stapedes are shown for *Heterocephalus*: the lower one comes from a neonate and has a small intercrural foramen and a cartilaginous head and neck (indicated by a dashed outline). Two stapedes are also shown for *Cryptomys*, in this case illustrating individual variation in the adult ossicle. Scale bar 2 mm. APSC = ampulla for posterior semicircular canal; ASC = anterior semicircular canal; C = cochlea; DMC = dorsal mastoid cavity; ED = bony tube for endolymphatic duct; ER = epitympanic recess; FP = footplate of stapes; HM = head of malleus; LA = lenticular apophysis of incus; LPI = long process of incus; LSC = lateral semicircular canal; MI = malleoincus; MM = manubrium of malleus; OW = oval window; PC = posterior crus of stapes; PD = bony tube for perilymphatic duct (canaliculus cochleae); PMC = posteromedial mastoid cavity; PSC = posterior semicircular canal; RW = round window; SPI = short process of incus; TC = tympanic cavity; TM = tympanic membrane; TT = tensor tympani tendon; V = vestibule of inner ear; VMC = ventral mastoid cavity.

The malleus and incus of the adult *Heterocephalus* are fused together (Figs 3A and 7A), although a trace of the articulation zone can be discerned. The malleus is the larger ossicle. It has a relatively short manubrium with prominent muscular and lateral processes. Internally, the manubrium consists of spongy bone surrounding large, open spaces, presumably vascular spaces (Fig 3B), which are not readily visible in a fresh specimen because of the periosteum covering the ossicle. The distal tip of the long process of the incus is similarly developed, but the bone is more compact elsewhere in the ossicular chain. The rounded malleus head, as its name suggests, does resemble the head of a hammer. It expands much more rostrally than caudally. Just below the head is a thin, bony lamina which represents a much abbreviated anterior process. The free edge of this lamina joins the bony ridge which supports the tympanic membrane and helps to divide the tympanic cavity from the epitympanic recess. The articulation here between malleus and skull appears to be ligamentous and it is very flexible. The incus has a long, narrow short process, which articulates with the skull at its tip, and a thicker long process which abruptly tapers and turns medially to support a very tiny lenticular apophysis, for articulation with the stapes. The connection between long process and lenticular apophysis is narrow and flexible (Fig 4A). The zone of fusion between malleus and incus extends half-way down the long process.

The stapes footplate is approximately oval in shape and averages 0.240 mm^2^ in area (n = 8). It is very thin centrally with a thickened ridge around its perimeter (Figs 4A and 7A). Its vestibular surface is quite flat. Instead of being stirrup-shaped with two crura, as would be typical for a placental mammal, a triangular, bony plate extends out of the middle of the tympanic side of the footplate. This very thin plate is strengthened by a low ridge on its dorsal aspect. Its apex expands slightly to form the head of the stapes, for articulation with the incus. There is no muscular process, consistent with the absence of a stapedius muscle.

### The bony labyrinth in *Heterocephalus*

**Fig 8:**
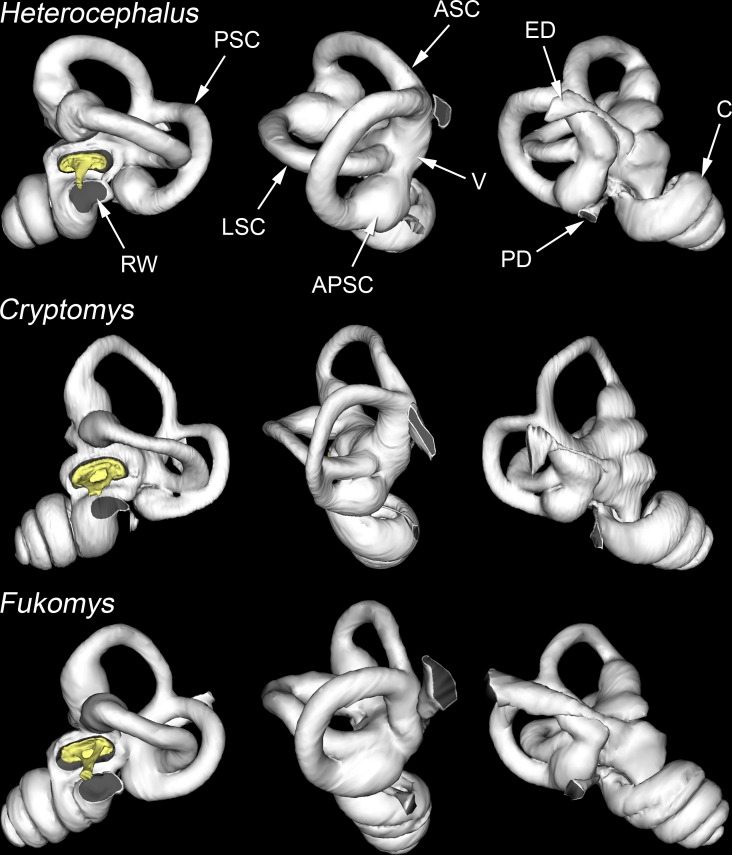
Bony labyrinths of three bathyergid species. WinSurf reconstructions of the left bony labyrinths of *Heterocephalus glaber*, *Cryptomys hottentotus* and *Fukomys micklemi*, seen from approximately lateral (left column), posterior (middle column) and medial (right column) views. Grey shading has been added to indicate the positions of the openings into the labyrinth. The stapedes are shown in yellow. Note that *Heterocephalus* has fewer cochlear turns than *Cryptomys* and *Fukomys*. The lateral semicircular canal of *Heterocephalus* reaches the vestibule directly, whereas in *Fukomys* it merges with the ampulla of the posterior canal; the condition in *Cryptomys* is intermediate. Not to scale. APSC = ampulla for posterior semicircular canal; ASC = anterior semicircular canal; C = cochlea; DMC = dorsal mastoid cavity; ED = bony tube for endolymphatic duct; ER = epitympanic recess; FP = footplate of stapes; HM = head of malleus; LA = lenticular apophysis of incus; LPI = long process of incus; LSC = lateral semicircular canal; MI = malleoincus; MM = manubrium of malleus; OW = oval window; PC = posterior crus of stapes; PD = bony tube for perilymphatic duct (canaliculus cochleae); PMC = posteromedial mastoid cavity; PSC = posterior semicircular canal; RW = round window; SPI = short process of incus; TC = tympanic cavity; TM = tympanic membrane; TT = tensor tympani tendon; V = vestibule of inner ear; VMC = ventral mastoid cavity.

The morphology of the inner ear was examined using CT reconstructions of the bony labyrinth (Fig 8). The semicircular canals are very wide relative to their length. The posterior end of the lateral canal opens directly into the vestibule, just above the ampulla of the posterior canal. The window framed by the arc of the posterior canal is divided by the lateral canal into a larger upper fenestration and a smaller lower one. An extension of the DMC passes through the upper fenestration in some specimens, while the PMC passes through the lower one (Fig 6). The narrow, orthogonal parts of the lateral and posterior canals approach each other extremely closely. In one 37-day and one adult specimen they were separated by a very thin, bony septum, whereas in other specimens the two canals were confluent at this point, although their contents were probably separated by soft tissue in life.

There are on average 2.74 cochlear turns; the length of the cochlear canal averages 5.79 mm (Table 3). The canaliculus cochleae, the very narrow channel for the perilymphatic duct, leaves the bony labyrinth just posterior to the round window, while the channel which carries the endolymphatic duct emerges from the vestibule at the base of the *crus commune* (the short canal segment shared by both anterior and posterior semicircular canals) and travels adjacent to the *crus commune*, widening just before it opens into the cranial cavity.

**Table 3:** Inner ear measurements, made from CT scan data.

Species	Cochlear turns	Cochlear canal length, mm	Cochlear *R*_base_, mm	Cochlear *R*_apex_, mm	Anterior SCC radius, mm	Posterior SCC radius, mm	Lateral SCC radius, mm	Mean internal diameter (all SCCs), mm
*Heterocephalus glaber*	2.74 ± 0.10, n = 6	5.79 ± 0.28, n = 6	0.50 ± 0.07, n = 6	0.13 ± 0.02, n = 6	0.78 ± 0.04, n = 4	0.69 ± 0.02, n = 4	0.66 ± 0.01, n = 4	0.36 ± 0.02, n = 4
*Bathyergus suillus*	3.90 ± 0.15, n = 2	16.80 ± 0.61, n = 2	0.90 ± 0.06, n = 2	0.31 ± 0.00, n = 2	N/A	N/A	N/A	N/A
*Cryptomys hottentotus*	3.53 ± 0.08, n = 3	10.04 ± 0.47, n = 3	0.76 ± 0.04, n = 3	0.13 ± 0.03, n = 3	1.04 ± 0.07, n = 3	0.85 ± 0.04, n = 3	0.82 ± 0.10, n = 3	0.33 ± 0.02, n = 2
*Fukomys micklemi*	3.84, n = 1	10.87, n = 1	0.57, n = 1	0.13, n = 1	0.94, n = 1	0.79, n = 1	0.83, n = 1	0.43, n = 1
*Georychus capensis*	3.50, n = 1	11.92, n = 1	0.76, n = 1	0.28, n = 1	1.18, n = 1	0.84, n = 1	0.86, n = 1	0.42, n = 1
*Heliophobius argenteocinereus*	3.02 ± 0.26, n = 2	10.23 ± 0.39, n = 2	0.84 ± 0.11, n = 2	0.17 ± 0.01, n = 2	N/A	N/A	N/A	N/A

Values cited are means ± standard deviation. The measurements of radii of curvature of the cochlear turns at base and apex (*R*_base_, *R*_apex_) follow the method given in [40]; measurements of semicircular canal (SCC) radii of curvature follow [41]. *Heterocephalus* measurements were from specimens over 100 days old only. The semicircular canals were not measured in *Bathyergus* and *Heliophobius* due to poor scan resolution.

### The ear region in very young *Heterocephalus* (<100 days old)

In the neonatal specimens (1–2 days old), the external ear canals were externally sealed and the middle ear cavities were filled with fluid. By 37 days, the external ears of the larger specimen were open, and the middle ear had begun to cavitate.

CT scans of the neonatal mole-rats showed that the tympanic bone remained unfused with the petrosal. The middle ear cavity had a very simple structure consisting of a small, rounded tympanic cavity with an epitympanic recess above. The mastoid cavities had not yet developed. The walls of the epitympanic recess had not yet ossified and the position of the tympanic membrane could not be discerned, so the recessus meatus could not be separated out from the middle ear component of the bulla. As a result, measurements of middle ear cavity volume (Table 2) could only be crude estimates. By 37 days, the boundaries of the middle ear cavity were clearer, although some small fenestrations between the bony elements remained. The air within the recessus meatus and tympanic cavity helped to define the position of the tympanic membrane in-between. Mastoid subcavities were still lacking, and middle ear morphology was overall very similar to that of the neonatal specimens.

The ossified parts of the malleus and incus were of the same shape and size in the neonates as in the older specimens. CT scans revealed them to be composed of a shell of cortical bone surrounding a large marrow space, although there were some bony trabeculae within the marrow space. Most of the manubrium, including the lateral process, remained cartilaginous, as did the head and part of the neck of the stapes. There was no lenticular apophysis of the incus and the position of the incudo-stapedial articulation was not clearly identifiable; instead, the long process of the incus simply tapered into a cartilage bar which was continuous with the bony body of the stapes. This bar proved to be flexible and tough. The malleus and incus were tightly articulated but could be separated with a scalpel blade.

The ossified part of the stapes body in the neonates was relatively squat and broad, but the bony part of the stapes footplate was smaller in area (0.166 mm^2^, n = 4) than in the adult specimens. In some specimens there was an unossified region at the centre of this ossicle, representing an intercrural foramen (Fig 7A). This was covered over with periosteum such that it was not patent. Histological sections of a neonatal *Heterocephalus* revealed the stapedio-vestibular articulation to be synovial (Fig 4B). The joint capsule extended between the stapes footplate and the rim of the oval window on both the tympanic and vestibular sides of the articulation, with an open space for synovial fluid in-between.

In the larger of the 37 day-old specimens, the manubrium was ossified but there was still cartilage between incus and stapes; the ossicles of the smaller specimen were less well-developed. The vascular cavities within the ossicles remained prominent, but malleus and incus were now fused. The stapedes lacked intercrural foramina.

The inner ears of the neonatal specimens were almost completely surrounded by a shell of bone. Although still poorly calcified, this allowed an accurate CT reconstruction of all parts except for the medial side of the lateral semicircular canal, where no ossification was visible and the boundaries of the canal had to be estimated. The inner ears were very similar in shape and volume to those of the adult specimens examined, but the volumes (Table 2) are overestimates because of the lack of ossification of internal structures such as the modiolus of the cochlea, which could not be subtracted from the total volume. The bone around the inner ear was more compact in the 37-day-old specimen and the boundaries of the lateral semicircular canal could be clearly distinguished (Fig 6). The internal structures of the cochlea were also better ossified. The reconstructed inner ear was of adult form.

### The ears of other bathyergid species

The middle ear cavities of the other bathyergids examined were more similar to each other than any of them were to that of *Heterocephalus* (Fig 5). The epitympanic recess was in all cases much larger, extending in a cranio-caudal direction. In *Heliophobius*, a septum just dorsal to the heads of the malleus and incus partially divided the epitympanic recess into rostral and caudal compartments; a less prominent version was present in *Georychus* and *Fukomys*, while in *Bathyergus* and *Cryptomys* the subcompartmentalization of the recess was irregular. The VMC was in all cases well-developed but in wide communication with the tympanic cavity, such that the two volumes could not be separated for individual measurement. The DMC, extending through the lateral semicircular canal, was always present but relatively small (Table 2). It was partially divided by a septum in all *Cryptomys* specimens and one *Bathyergus*. Posteromedial mastoid cavities were lacking. Three or four small septa were present ventral to the inner ear as it projected into the tympanic cavity.

The mallei and incudes were similar in all bathyergids, although the shapes of the malleus heads differed (Fig 7), as did the width of the manubrial blades, as seen from rostrally (widest in *Bathyergus*). The zone of fusion between malleus and incus always extended down the incudal long process but it was not continuous: the two ossicles separated slightly somewhere in the middle of the articulating region, to a variable extent between individuals. The manubria and incudal long processes were made of compact bone, as opposed to the spongy structure found in *Heterocephalus*. The stapedes all possessed intercrural foramina, the size of which varied among *Cryptomys* specimens (Fig 7F). The gap separating the footplate from the rim of the oval window was particularly wide in this species. It was not possible to discern the nature of the articulation in most of the CT scans, but in one *Fukomys* and one *Cryptomys* specimen a synovial cavity could be seen within the zone of articulation. All species showed signs of a tensor tympani muscle, but no stapedius was found.

The number of cochlear turns in one *Heliophobius* specimen was in the *Heterocephalus* range, but in this animal both left and right cochleae were unusually loosely coiled: it looked like the apical coils had failed to develop properly. In all other bathyergids, including the other *Heliophobius*, the numbers of turns were greater (Table 3), reaching a maximum value of 4.00 in one specimen of *Bathyergus*. How the posterior limb of the lateral semicircular canal reached the rest of the inner ear also varied between species. In *Heliophobius* it entered the vestibule directly, as in *Heterocephalus*. In *Fukomys* (Fig 8) and *Bathyergus* it joined the ampulla of the posterior canal. The morphology of *Georychus* was intermediate: although the lateral canal converged with the posterior ampulla before reaching the vestibule, the two remained externally demarcated. The three *Cryptomys* specimens varied between the *Heterocephalus* and *Georychus* conditions.

## Discussion

### Ctenohystrica ear features in bathyergids

The Ctenohystrica rodent group, to which the bathyergids belong, has some very characteristic middle ear features [43, 44]. These include a malleus with a ‘bullet-shaped’ head and a very reduced anterior process, malleo-incudal fusion and a relatively loose stapedio-vestibular articulation, which has been found to be synovial rather than fibrous in the species examined. Certain species such as chinchillas have very capacious middle ear cavities. Loose articulations and large cavities increase middle ear compliance, which aids low-frequency sound transmission. This is thought to be advantageous to arid-region species because low-frequency sound propagates best in such environments [45, 46]. It is therefore possible that the ancestral Ctenohystrica rodents evolved in an arid habitat. However, ‘low-frequency’ ears are also considered to be advantageous in subterranean environments (see Introduction). It has been noted that certain features of Ctenohystrica rodents, including their generally blunt body-shapes and small pinnae and tails, are also characteristic of subterranean mammals, while a unique carpal bone found in the hystricognath subgroup is considered an adaptation to digging [47]. This raises the possibility that the ancestral Ctenohystrica rodents were fossorial. Whether or not this was the case, it is no surprise that tuco-tucos (*Ctenomys* species: [24, 48]) and the coruro (*Spalacopus*: [22]), which are subterranean, retain typical, low-frequency, Ctenohystrica-type ears. Our findings, which confirm and extend previous descriptions of the ear in the Bathyergidae (see Introduction), lead to the conclusion that the ears of this family of mole-rats also retain many ancestral Ctenohystrica features. However, bathyergids are unique in that their malleo-incudal fusion zone extends down the long process of the incus [36].

The tensor tympani muscle can be well-developed in Ctenohystrica rodents, but the stapedius is typically reduced or absent [43, 44]. In the present study, a stapedius was not found in any bathyergid, consistent with several previous descriptions [20, 34, 35, 49]. However, some other reports suggest that a small stapedius may be retained in some individual mole-rats [26, 27, 36]

Mason [43] hypothesized that a very compliant stapedio-vestibular articulation could not be stiffened to a functionally relevant extent by the stapedius muscle. This may have led that muscle to degenerate, while a compensatory role of the tensor tympani could in principle be augmented by malleo-incudal fusion. These Ctenohystrica characteristics may therefore be functionally linked. Not only is the stapedio-vestibular articulation synovial in at least some bathyergids (this could not be assessed in all species) but the gap between stapes footplate and oval window is unusually wide (Fig 4A), which is expected to increase compliance still further. A very wide gap has previously been documented in the spalacid mole-rat *Spalax* [23] and the gerbil *Gerbillurus* [42].

Among the Ctenohystrica, the cochleae of *Ctenodactylus* and *Petromus* have 3.5 turns [34], and a ‘high-spiralled’ cochlea with a similarly large number of turns is also characteristic of caviomorph rodents [22, 41, 50, 51]. We found up to 4 cochlear turns in the bathyergids we examined (Table 3), while Müller et al. [30] found 4 to 4.5 turns in *Fukomys anselli*. High-spiralled cochleae therefore appear to be widespread within the Ctenohystrica, and may well be primitive both for this group and for the Bathyergidae.

### Unusual features of the *Heterocephalus* ear

Although bathyergid ears have many features in common, the naked mole-rat stands apart from the others in terms of the smaller relative size of its epitympanic recess, the elaboration of its mastoid subcavities (Figs 5 and 6), the spongy bone of its ossicular processes (Fig 3B) and the structure of its stapes, which lacks an intercrural foramen in the adult (Fig 7A). Its inner ear is unusual in its low number of cochlear turns (Fig 8) and, probably related to this, its short cochlear canal length (Table 3).

A ‘columelliform’ stapedial morphology was found in one of two specimens of *Georychus* examined by Burda et al. [20], but not in the specimen that we examined. We are unaware of any other report of a columelliform stapes in a rodent but they are found in certain other mammals including monotremes, some marsupials, pangolins, some edentates and many aquatic species [36, 52, 53]. The existence of a tiny foramen in some neonatal *Heterocephalus* specimens (Fig 7A) may reflect the presence of a stapedial artery in the embryo. This artery regresses in many mammals, typically leaving an empty intercrural foramen [52] although a bony spicule passes through it in caviomorph rodents [54]. The arch made by the stapedial crura is thought to be mechanically advantageous in resisting bending [53], and the relative development of the stapes crura may be influenced by the pull of the stapedius muscle [55]. *Heterocephalus* lacks this muscle, but so too do many other Ctenohystrica rodents which nonetheless retain normally-shaped stapedes, including other bathyergids.

Molecular phylogenetic studies suggest that *Heterocephalus* forms a basal outgroup to the rest of the Bathyergidae [1, 56]. This genus is sometimes placed in a separate subfamily, the Heterocephalinae, with the other genera forming the Bathyerginae [57]. It is therefore possible that *Heterocephalus* retains the primitive ear characteristics for its family, while the bathyergines are derived. However, the bathyergines are more similar to other Ctenohystrica species in terms of their ossicular process ossification, stapedial morphology and cochlear coiling. This suggests that, in these respects at least, it is *Heterocephalus* which shows the derived condition. It is not clear what the ancestral middle ear cavity morphology would be for the Ctenohystrica because this varies so much among different rodent species, even within the same family [23, 34, 42]. *Heterocephalus* is the smallest bathyergid: although cochlear coiling is unrelated to body size among mammals in general [41], it is possible that within the Bathyergidae the number of turns is somehow restricted by size, and that size affects middle ear cavity structure.

These unusual characteristics of the naked mole-rat ear are discussed again later, in the context of possible degeneration of its peripheral auditory system.

### Post-natal ear development in *Heterocephalus*

At birth, the naked mole-rat has a simple middle-ear cavity structure based on a tympanic cavity and epitympanic recess only. The posterior part of the tympanic cavity goes on to invade the area around the semi-circular canals, creating the three mastoid subcavities (Fig 6). Post-natal expansion of the middle ear cavity into the mastoid region has been documented in the kangaroo-rat *Dipodomys* [58], and a general increase in middle ear cavity volume has been found in murid rodents [59–61]. Subcavities were not distinguished in the murid studies. The extent of expansion of the mastoid subcavities varies between individual naked mole-rats in a way that does not relate closely to age (Table 2), and septa were also found to be variably developed. Intraspecific variation in mastoid cavity structure has been noted in gerbils [62] and dormice [63], but in these studies the ages of the specimens were not recorded so the differences could, in principle, have been ontogenetic.

The malleus and incus of the naked mole-rat appear to be better-developed at birth than those of other altricial rodents [58, 59, 61, 64], but the manubrium of the malleus and distal long process of the incus remain cartilaginous. Once ossified, these processes are composed of spongy bone with large vascular spaces, and they were not remodelled into compact bone even in our oldest specimen (65 months). Stapes structure changes significantly post-natally: the head and neck ossify, the bony footplate expands slightly and the body is slimmed down. During this process the intercrural foramen, if still present, is lost. Substantial postnatal remodelling of the stapes has also been observed in the rat, kangaroo rat and rabbit, but in these animals the intercrural foramen widens [58, 59, 65].

Unlike the middle ear cavity, the inner ear of *Heterocephalus* had achieved its adult dimensions in the neonatal specimens. It seems to be a common trend among mammals that the bony labyrinth changes little, post-ossification (see e.g. [65–67]). The membranous semicircular canals of *Fukomys* mole-rats have also been found not to expand postnatally [31].

### The effect of the middle ear on hearing range

An electrical analogue model of the middle ear constructed by Hemilä et al. [68] proved to be surprisingly accurate in predicting high-frequency hearing limits in a wide range of mammals. The three anatomical parameters used in the model were tympanic membrane area, ossicular mass and a measure of malleus length. Mason [21] put measurements from *Heterocephalus* (taken from [38]) into this model and predicted a high-frequency hearing limit of 99.7 kHz at 60 dB SPL, a high value reflecting the small size of the ear. This prediction greatly exceeds the mole-rat’s behavioural hearing limit of 12.8 kHz [9]. These results indicate that the gross structural dimensions of the *Heterocephalus* middle ear should be adequate for the transmission of high, ultrasonic frequencies, suggesting that the limitation to high-frequency hearing in this animal lies elsewhere.

In terms of low-frequency hearing, middle ear cavity volume is particularly important to small mammals [25]. This is because cavity compliance, which is proportional to volume, often dominates overall middle ear compliance in these animals [69], and compliance determines sound transmission at low frequencies. A laboratory rat, which is not a low-frequency specialist, has a middle ear cavity volume around 60 mm^3^ [59]. The spalacid mole-rat *Spalax ehrenbergi* has a middle ear cavity which is of moderate size (around 100 mm^3^: [23]). The hearing of *Spalax* is more acute than that of rats at frequencies below around 1 kHz, but the low-frequency hearing of arid-region rodents such as gerbils, kangaroo rats and chinchillas tends to be better still [9, 13, 70]. These species have much more capacious middle ear cavities: gerbils have cavity volumes ranging from 145–1017 mm^3^ [42, 62], kangaroo rats of the genus *Dipodomys* have cavity volumes from 430–1410 mm^3^ [71], while the cavity volume of the chinchilla can exceed 1500 mm^3^ [54, 72].

Despite the expansion of its middle ear cavity into the mastoid region, the total middle ear cavity volume in *Heterocephalus* is only around 25–30 mm^3^ (Table 2), which is under the value expected for a terrestrial mammal of its body size [21]. *Bathyergus suillus*, which is ten times the weight of the naked mole-rat, has a cavity volume around 440 mm^3^, while the other bathyergids examined are of intermediate size and have intermediate cavity volumes (Table 2). Assuming that cavity compliance is limiting, we would expect low-frequency hearing limits to correlate inversely with body size in this group, but the data required to test this hypothesis are lacking. Although postnatal expansion of the cavity into the mastoid region in bathyergids might be the result of selective pressure to improve low-frequency sound transmission, the small middle ear cavity of the naked mole-rat does not suggest that its low-frequency hearing could be particularly acute. This conclusion is borne out by behavioural hearing studies, which have found the thresholds of *Heterocephalus* at frequencies under 1 kHz to be somewhere between those of laboratory rats and the arid-region rodents mentioned above [9]. The fact that the rat has a larger total cavity volume than *Heterocephalus* but poorer low-frequency hearing suggests that the rat’s much stiffer ossicular suspension dominates its middle ear compliance.

Subcompartmentalisation of the middle ear is not expected to have a significant impact at low frequencies but might result in mid-frequency resonances. One significant resonance has been observed and modelled in the cat, the middle ear of which is partially divided into two large subcavities [73], and more resonances have been found in the middle ear response of the chinchilla, which has many smaller subcavities [45]. However, no such resonances were observed in the ear of the gerbil *Meriones* [69], which has a similar cavity structure to bathyergids [42]. While it is possible that the partial divisions in gerbil and bathyergid middle ears serve purely structural purposes, further experimental work is needed to confirm that they have no significant functional effect.

### Bone conduction

Foot-drumming and stomping are used for intraspecific communication in several species of bathyergids, but these behaviours have not been observed in *Heterocephalus* or *Heliophobius* (see [74] for a review). Ground vibrations generated in this way could be detected by the somatosensory system, or alternatively by the ear through a form of bone conduction. Certain species of golden moles [75] and talpid moles [76] have relatively enormous mallei which likely underpin a form of inertial bone conduction. The mole-rat *Spalax* lacks notably enlarged ossicles, but other features of its ear region may augment alternative modes of bone-conducted hearing [23, 77].

The malleoincus unit of *Heterocephalus* weighs around 0.47 mg (n = 2), while its stapes weighs around 0.026 mg (n = 1) [38]. These values are slightly smaller than expected for its body mass [21], which is quite unlike the situation in golden moles. *Heterocephalus* shares certain features of its middle ear with that of *Spalax*, however, notably a loose articulation between malleus and skull, a flexible incudo-stapedial articulation and a particularly wide annular ligament of the stapes. It has been suggested that these features may reduce constraint to the point where the ossicular chain could vibrate in response to skull vibrations [23]. However, any role of this in promoting bone conduction will be limited by the small mass of the ossicles in these animals.

### Inner ear structure and function

**Fig 9:**
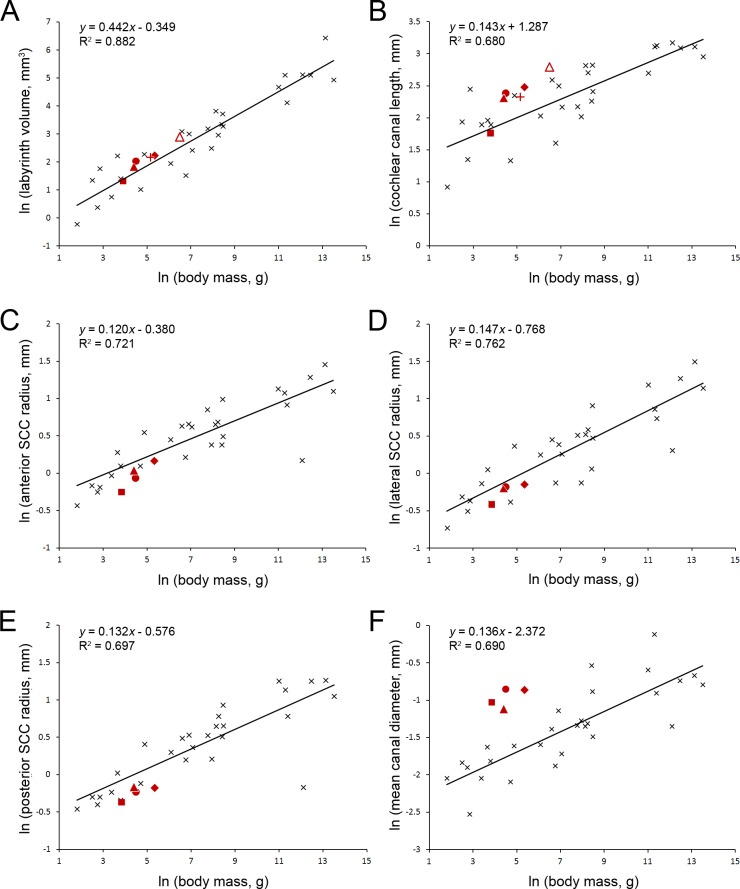
Relationships between inner ear measurements and body mass. Relationships between the natural logarithms of inner ear measurements and body mass. Plot A considers the total volume of the bony labyrinth. Plot B considers the length of the cochlear canal. Plots C, D and E consider the radii of curvature of the anterior semicircular canal (SCC), lateral semicircular canal and posterior semicircular canal respectively. Plot F shows the mean cross-sectional diameter of all three semicircular canals. In each plot, data for 28 extant mammalian species (black crosses) were taken from Ekdale (2013), and a least squares regression line fitted as in that paper. Data-points representing mean values for bathyergids were superimposed as red symbols: *Heterocephalus* individuals over 100 days old (square), *Bathyergus* (open triangle), *Cryptomys* (solid triangle), *Fukomys* (circle), *Georychus* (diamond) and *Heliophobius* (plus). Body mass data, where available, were taken from Table 1 for the individual animals concerned. Unrecorded body masses were estimated as follows: *Fukomys* 89 g, a mean value for animals from the same laboratory colony [78]; *Cryptomys* 82 g, *Georychus* 211 g [79]. Inner ear data were taken from Tables 2 and 3.

Ekdale [41] found that bony labyrinth volume and cochlear canal length both correlate with body mass among mammals in general. The volumes measured here in bathyergids fit his mammalian trend-line well (Fig 9A). The cochlear canal length of *Heterocephalus* also falls very close to Ekdale’s trend-line, but canal lengths in the other mole-rats examined (the bathyergines) fall above it (Fig 9B). This likely relates to increased cochlear coiling in the bathyergines. However, cochlear canal length should approximate basilar membrane length, and for the five species in common, Ekdale’s cochlear canal length measurements average 65% of the basilar membrane length measurements collected from the literature by Manoussaki et al. [40]. If Ekdale’s values are all revised upwards, the cochlear canal lengths of the bathyergines would be very close to expected, while that of *Heterocephalus* would be shorter than expected for its body size.

West [80] identified strong correlations between upper and lower hearing limits and inner ear dimensions, for mammals in general:

$$log_{10}\left(LFL\right)=1.76-1.66log_{10}\left(BML\times CT\right)$$

$$log_{10}\left(HFL\right)=2.42-0.994log_{10}\left(BML/CT\right)$$

where LFL = low-frequency hearing limit at 60 dB SPL (kHz), HFL = high-frequency hearing limit at 60 dB SPL (kHz), BML = basilar membrane length (mm) and CT = number of cochlear turns. Using these equations, and using cochlear canal length as an estimate of basilar membrane length, one can predict a low-frequency hearing limit of 585 Hz and a high-frequency limit of 125 kHz in *Heterocephalus* (Table 4). These values compare poorly with the naked mole-rat’s behavioural hearing limits of 65 Hz and 12.8 kHz respectively [9]. Manoussaki et al. [40] found a relationship between the radii of curvature at the cochlear base and apex and low-frequency hearing limits in mammals:

$$LFL=1.507\;exp\left[\text{-}0.578\left(R_{base}/R_{apex}-1\right)\right]$$

where *R*_base_ is the radius of curvature of the cochlear basal turn (mm) and *R*_apex_ is the radius of curvature of the cochlear apical turn (mm). Using this equation, the low-frequency hearing limit of *Heterocephalus* should be 291 Hz (Table 4), which is still much higher than the observed value. These results suggest that normal mammalian structure-function relationships do not hold for *Heterocephalus*, which has better low-frequency hearing but much poorer high-frequency hearing than expected. Audiograms are not available for the other bathyergids examined here, but predictions from the three equations are included in Table 4 for comparative purposes.

**Table 4:** Predictions of hearing limits in bathyergid mole-rats.

Species	LFL from Manoussaki et al. (2008) equation, kHz	LFL from West (1985) equation, kHz	HFL from West (1985) equation, kHz
*Heterocephalus glaber*	0.291	0.585	125
*Bathyergus suillus*	0.502	0.056	61.6
*Cryptomys hottentotus*	0.092	0.154	93.1
*Fukomys micklemi*	0.213	0.117	93.5
*Georychus capensis*	0.559	0.118	77.8
*Heliophobius argenteocinereus*	0.154	0.194	78.2

LFL = 60 dB SPL low-frequency hearing limit; HFL = 60 dB SPL high-frequency hearing limit. Mean values taken from Table 3 were used in the calculations; the length of the cochlear canal was used as an estimate of basilar membrane length. A comparison of the predictions for *Heterocephalus* with audiogram data suggests that there is little reason to be confident about these values (see text).

Soft tissue structures of the cochlea could not be observed in our CT scans, but were described in *Fukomys anselli* by Müller et al. [30]. Unusual features included a scala tympani and helicotrema of very small cross-sectional areas, and a very low spiral ganglion cell density. The basilar membrane is also of almost constant width and thickness over nearly half of its length, and frequencies between 0.6 and 1 kHz were found to be tonotopically over-represented. However, there is no evidence for the particularly sharp tuning which would be expected of a low-frequency “acoustic fovea” [81]. Müller et al. [30] noted that *F*. *anselli* has a more limited range of hearing than predicted from anatomical correlations.

Turning to the vestibular system, the posterior limb of the lateral semicircular canal may enter the vestibule directly (e.g. in *Heterocephalus*), merge with the posterior ampulla (e.g. in *Fukomys*) or show an intermediate morphology (Fig 8). Direct entry into the vestibule was found in the mouse (*Mus*) and guinea pig (*Cavia*), and was reconstructed as primitive both for Rodentia and placental mammals as a whole [41]. This may well be so, but the diversity of phenotypes found here in just one family illustrates the need for caution in the interpretation of the direction of inner ear evolution based on the sampling of a few, isolated species. The nature of the entry of the lateral canal into the vestibule influences whether there is a space between lateral and posterior canals through which a posteromedial mastoid cavity can emerge, but the functional implications of these differences in lateral canal morphology are otherwise unknown.

One of the most striking features of the bony labyrinth of bathyergids is the ‘short and fat’ appearance of the semicircular canals (Fig 8). Our measurements (Table 3) may be compared to values for a wide range of mammals tabulated by Ekdale [41]. The radii of curvature of each of the semicircular canals of bathyergids are smaller than expected for their body masses (Fig 9C,9D and 9E), but the canal cross-sectional diameters are substantially greater than expected (Fig 9F). The ratios of canal diameter to radius of curvature are as a result very high. From Ekdale’s data, only the pangolin *Manis* and an extinct elephantimorph of uncertain identity have canals of similar proportions. Among subterranean mammals, data tabulated by Crumpton et al. [82] show that certain golden moles and the marsupial mole *Notoryctes* can be added to that list, while the membranous semicircular canals of the spalacid mole-rat *Spalax* have similar dimensions to those of *Fukomys* [33]. ‘Short and fat’ canals have evidently evolved several times in parallel among subterranean mammals, although they are not found in all species.

According to the theoretical model developed by Oman et al. [83], mechanical sensitivity of a given semicircular canal, in terms of displacement of the cupula per unit head angular velocity, will be proportional to an ‘average radius of curvature’ of the entire fluid streamline and to its average squared cross-sectional area, weighted according to deviations from perfect circularity. These measurements should include not just the narrow section of duct but the entire circuit, including ampulla and utricular cavity, and should be based on the membranous labyrinth rather than the bony labyrinth considered here. The Oman et al. model has been used to predict that the sensitivities of the wide semicircular canals of bathyergid and spalacid mole-rats are enhanced in comparison to those of murid rats and guinea pigs [32, 33].

### Does the ear of *Heterocephalus* show signs of degeneration?

Anatomical measurements of peripheral auditory structures can be used to predict the upper and lower hearing limits of mammals. In the cases of the Hemilä et al. [68] model and the West [80] correlation equations, smaller ears generate higher-frequency predictions. Smaller animals (with smaller ears) do indeed tend to have hearing ranges shifted to higher frequencies, but the predictions are strikingly inaccurate when it comes to the naked mole-rat. *Heterocephalus* has an ear which, from its small dimensions, would appear suitable for the transmission of high, ultrasonic frequencies, but a hearing range restricted to very low frequencies. It has been suggested that the central auditory system is responsible for the high thresholds and rudimentary localisation abilities observed in this animal [9, 10], in which case the structure of its peripheral auditory system might give no indication of its hearing limitations. However, we have identified some features of the ear of the naked mole-rat which might contribute to poor hearing and/or could be considered degenerate:

1. *Heterocephalus* lacks pinnae to channel sound into the ear and provide ‘pinna cues’ to aid localisation [9]. Its external ear canals are very narrow and semi-occluded (Fig 1B), which will reduce the sound energy reaching the tympanic membrane. Cerumen has been found in the bony external meatus of several other bathyergid species [26].
2. The manubrium of the malleus and the distal tip of the incudal long process have the spongy and poorly-ossified appearance of newly-developed bone, even in adult animals (Fig 3B).
3. The columelliform stapes has a thin, flexible body which appears to lack the structural stiffness typical of this ossicle. It articulates with a tiny lenticular apophysis mounted on a weakly-developed long process of the incus (Fig 4A). Increased flexibility within the ossicular chain can result in reduced sound energy transmission at high frequencies [84].
4. Reduced cochlear coiling and cochlear canal length in *Heterocephalus* might conceivably represent a degenerate feature, if having a ‘high-spiralled’ cochlea was the ancestral condition for bathyergids.

Like that of *Heterocephalus*, the hearing range of *Fukomys anselli* is known to be very limited [11, 12], and it may be that hearing is poor in all members of the Bathyergidae. Points 2 to 4 above suggest that the hearing of *Heterocephalus* might be worse than that of the other bathyergids examined. However, these anatomical differences could be influenced by the small size of the naked mole-rat, its phylogenetic position as the most basal offshoot of the family or the fact that our specimens came from laboratory colonies rather than the wild. Our specimens were also relatively young for naked mole-rats, albeit of comparable ages to the approximately one-year-old animals which had their hearing tested by Heffner & Heffner [9].

Increased intraspecific variability is a possible indicator of a lack of selective pressure constraining ear structure. If the variation in the extent of pneumatisation of the mastoid region among adult *Heterocephalus* proves to exceed that found in other small mammals, this feature could be added to the list above. Our animals were from captive colonies, but the differences in stapes structure found here in *Cryptomys* specimens and the differences in cochlear structure in *Heliophobius* specimens, all collected from the wild, could be interpreted along similar lines. Significant intraspecific variability in stapes structure has previously been recorded in *Georychus* [20] and in the *Spalax ehrenbergi* superspecies [85, 86]; *Spalax* is known to have ‘degenerate’ hearing similar to that of *Heterocephalus* [13]. We did not find the same level of intraspecific variability in all middle and inner ear structures, however, which could indicate that some features retain more functional importance than others.

Although the anatomical features noted above are suggestive of degeneration, in accordance with what is known about the hearing of the naked mole-rat, none are conclusive. Given the dangers of *post-hoc* interpretations of this nature, experimental studies are necessary to test these ideas.

## Supporting Information

S1 Table Middle and inner ear measurements. Worksheet 1 contains the measurements made on individual animals from which mean values were calculated. Worksheet 2 contains the *x*,*y*,*z* coordinates of points along the cochlear ducts, which were used to calculate some of the inner ear parameters represented in Table 3.
(XLSX)
